# Celebrating a breakthrough: FDA greenlights TriClip G4 for tricuspid regurgitation: an editorial

**DOI:** 10.1097/JS9.0000000000001628

**Published:** 2024-05-17

**Authors:** Aman Goyal, Samia A. Sulaiman, Vidhi Pancholi, Hritvik Jain, Mohamed Daoud, Amir H. Sohail

**Affiliations:** a Department of Internal Medicine, Seth GS Medical College and KEM Hospital, Mumbai; b Department of Internal Medicine, All India Institute of Medical Sciences-Jodhpur, Rajasthan, India; c School of Medicine, University of Jordan, Amman, Jordan; d Department of Internal Medicine, Bogomolets National Medical University, Kyiv, Ukraine; e Department of Surgery, University of New Mexico Health Sciences, Albuquerque, New Mexico, USA

The U.S. Food and Drug Administration (FDA) recently approved EVOQUE and Abbott’s TriClip G4 in October 2023 and February 2024, respectively, for the treatment of tricuspid regurgitation (TR), both of which offer groundbreaking developments in transcatheter treatment modalities for this condition^1,2^. TR, often known as the ‘forgotten valve disease’ is a highly prevalent condition and encompasses primary and secondary etiologies, with the latter being more prevalent^3^. Primary TR occurs due to intrinsic abnormalities originating in the tricuspid valvular apparatus, whereas secondary TR results from right atrial or ventricular dilatation, resulting in regurgitation^3,4^. According to Mulla and colleagues, patients with TR commonly present with features of right-sided heart failure, which may include ascites, peripheral edema, and hepatosplenomegaly, although this is more often found as an incidental echocardiographic finding^3–5^.

Ricci *et al*.^5^ demonstrated that TR is common and is frequently associated with right ventricular dysfunction, pulmonary hypertension, left heart disease, long-standing atrial fibrillation, the presence of a cardiac device’s leads crossing the valve, and aging. Due to its association with aging, evidence shows that TR is found in ~1 in 25 elderly individuals and is more common in women than in men^4,5^. Condello *et al*.^4^ also highlighted that up to half of the patients with severe mitral regurgitation and one-quarter of the patients with severe aortic stenosis develop at least moderate and severe TR. This observation holds significant prognostic implications, as the coexistence of TR and other cardiac pathologies frequently exacerbates the overall clinical prognosis, irrespective of the primary etiology^4^. Therefore, due to the increased risk of morbidity and mortality with TR, effectively managing and addressing the condition is of significant clinical importance, which highlights how the recent approval of EVOQUE and TriClip G4 both contribute to alleviating the burden on patients and the healthcare system. Current diagnostic criteria include Doppler Echocardiography as the main modality for accurately measuring the flow velocity of regurgitant blood flow and the systolic pressure in the right ventricle, although chest radiography, serum testing, electrocardiography, cardiac catheterization, and cardiac magnetic resonance could also be considered additional tests beneficial in diagnosis^3^. The gold standard for treating severe TR remains surgical repair, whereas tricuspid valve replacement is considered in cases where repair is not feasible^3–5^. In asymptomatic patients with severe TR and non-dilated right ventricle and in symptomatic patients with severe secondary TR and either severe right ventricular dysfunction or irreversible pulmonary hypertension, conservative management with medical treatment is recommended^5^. However, guidelines regarding the optimal timing for isolated tricuspid valve surgery remain conflicting^3^.

The EVOQUE consists of a tri-leaflet bovine pericardial tissue valve, a nitinol frame, and a fabric skirt. It is designed to replace the tricuspid valve and has demonstrated procedural success in 94% of patients^1,4^. The TRISCEND study (Edwards EVOQUE Tricuspid Valve Replacement: Investigation of Safety and Clinical Efficacy after Replacement of Tricuspid Valve with Transcatheter Device) was performed by Kodali *et al*.^6^. This study aimed to assess the safety and efficacy of transfemoral transcatheter tricuspid valve replacement in individuals with clinically severe TR and heightened surgical risk. This was a prospective, single-arm, multicenter trial involving 56 patients^6^. At the 30-day follow-up mark, all patients had at least 1-grade reduction in the severity of their TR, and 98.1% had at least 2-grade reduction, demonstrating the efficacy of the EVOQUE system. The Kansas City Cardiomyopathy Questionnaire (KCCQ) score of the patients also improved significantly in studies conducted by Kodali *et al*.^6^ and Arnold *et al*.^7^. The trial described major adverse events (MAEs) as cardiovascular mortality, myocardial infarction, stroke, renal complications necessitating unplanned dialysis or renal replacement therapy, severe bleeding classified as fatal, life-threatening, extensive, or major bleeding, nonelective thoracic reintervention, major complications at the access site and vascular level, major cardiac structural complications, and device-related pulmonary embolism. A total of 15 patients, accounting for 26.8% of the sample, had MAEs. Among them, one patient had cardiovascular mortality (1.8%), two patients underwent nonelective tricuspid valve re-interventions, one patient experienced a major access site or vascular problem, and 15 patients experienced serious bleeding events that were either non-life-threatening or fatal. There were no instances of myocardial infarction, stroke, renal failure necessitating dialysis, significant heart structural problems, or device-related pulmonary embolism.

TriClip offers new steerable catheters suited for the right side of the heart^4^. Interestingly, although the end-diastolic and end-systolic areas of the right ventricle showed significant improvement, decreasing from 33.1±7.2 cm^2^ to 23.4±7.1 cm^2^ (*P*<0.001) and from 20.5±5.1 cm^2^ to 17.6±5.8 cm^2^ (*P*=0.001), respectively, there was a decline in right ventricular function from baseline to 30 days, as evidenced by a decrease in tricuspid annular plane systolic excursion (14.9±3.9 mm to 13.0±3.19 mm; *P*=0.035) and right ventricular fractional area change (37.6±9.3% to 24.8±9.9%; *P*<0.001)^6^.

Sorajja *et al*. conducted a prospective randomized experiment to evaluate the efficacy of percutaneous transcatheter edge-to-edge repair (TEER), which is a transvenous approach where a clip is deployed to hold the tricuspid valve’s leaflets together and reduce TR without cardiac surgery or cardiopulmonary bypass, in treating severe TR using the TriClip device. A total of 65 sites in the United States, Canada, and Europe recruited patients with symptomatic severe TR. These patients were randomly divided into two groups, with each group receiving either TEER or medical therapy^8^. A total of 350 patients were included, with 175 patients assigned to each group. Despite both groups reporting similar improvements in terms of all-cause mortality, tricuspid valve surgery, or heart failure hospitalizations, TEER with TriClip demonstrated more favorable enhancements in patients’ quality of life, as assessed by the KCCQ score at 1 year, thus favoring its use^8^. Furthermore, over 98% of patients were free from MAEs (defined as death from cardiovascular causes, new-onset kidney failure, endocarditis treated with surgery, and nonelective cardiovascular surgery for a TriClip device-related adverse event in the TRILUMINATE trial) after 30 days, which offers insights into the safety of TriClip interventions^8^. Nevertheless, further research into the long-term outcomes after TriClip and population-specific studies are still necessary, as Condello *et al*.^4^ noted that entanglement with subvalvular structures (chordae tendineae) and multiple attempts due to frequent large coaptation gaps could potentially pose challenges in TriClip interventions.

The current editorial highlights the need for continued clinical trials to elucidate the long-term benefits and risks, durability, and comparative effectiveness of both TEER interventions, which could improve the overall benefits for patients with TR. Patient experience and ongoing advancements in imaging modalities, device technologies, and procedural techniques have the potential to optimize treatment strategies. Further research must also focus on the epidemiology of TR among the general population and in different patient populations, as well as the comparative effects between novel, approved TEER modalities and traditional medical treatment, which could provide comprehensive insights into the treatment of TR. Overall, FDA approvals of the EVOQUE and TriClip G4 systems represent transformative advances in the management of TR, relevant to improving the symptoms of patients and their prognoses, as well as providing insights into future clinical practice regarding TR management. Through these systems, earlier management of tricuspid valve diseases can facilitate further optimization of results.

## Ethical approval

Due to the nature of this paper, it is exempt from ethical approval.

## Consent

Due to the nature of this paper, it is exempt from consent.

## Sources of funding

The authors did not receive any funding.

## Author contribution

A.G.: study concept, design, data collection, analysis, interpretation, and writing the paper; S.A.S. and A.H.S.: study concept, data collection, analysis, interpretation, and writing the paper; M.D.: writing the paper.

## Conflicts of interest disclosure

The authors report no financial relationships or conflicts of interest regarding the content herein.

## Research registration unique identifying number (UIN)

Due to the nature of this paper, it is exempt from requiring a UIN.

## Guarantor

Mohamed Daoud, MD; Department of Internal Medicine, Bogomolets National Medical University, Kyiv, Ukraine; Mailing address: Bogomolets National Medical University, Peremohy Ave, 34, Kyiv 02000, Ukraine; E-mail: drmohameddaoudmd@gmail.com.

## Data availability statement

Data sharing is not applicable to this article.

## Provenance and peer review

Not commissioned, externally peer-reviewed.
